# Laboratory Evaluation of Flight Capacities of *Aedes japonicus* (Diptera: Culicidae) Using a Flight Mill Device

**DOI:** 10.1093/jisesa/ieab093

**Published:** 2021-12-04

**Authors:** Eva Krupa, Alexa-Lou Gréhal, Jérémy Esnault, Christelle Bender, Bruno Mathieu

**Affiliations:** 1 Institut de Parasitologie et Pathologie Tropicale, UR7292 Dynamique des interactions hôte pathogène, Fédération de Médecine Translationnelle, Université de Strasbourg, F-67000, Strasbourg, France; 2 Syndicat de Lutte contre les Moustiques du Bas-Rhin (SLM67), F-67630, Lauterbourg, France

**Keywords:** dispersion, mosquito, vector, age

## Abstract

Dispersion expands the distribution of invasive species and as such, it is a key factor of the colonization process. *Aedes japonicus japonicus* (Theobald, 1901) is an invasive species of mosquito and a vector of various viruses. It was detected in the northeast of France in 2014. The population of this species can expand its distribution by several kilometers per year. However, though flight capacities play an active part in the dispersion of *Ae. japonicus*, they remain unknown for this species. In this study, we investigated the flight capacities of *Ae. japonicus* in a laboratory setting using the flight mill technique. We evaluated the influence of age on flight. We recorded videos of individual flights with a camera mounted on Raspberry Pi. We extracted data on distance, duration, and speed of flight using the Toxtrac and Boris software. Our analysis showed a median flight distance of 438 m with a maximum of 11,466 m. Strong flyers, which represented 10% of the females tested, flew more than 6,115 m during 4 h and 28 min at a speed of 1.7 km per h. As suspected, *Ae. japonicus* is a stronger flyer than the other invasive species *Aedes albopictus* (Skuse, 1894) (Diptera: Culicidae). To our knowledge, this is the first flight mill study conducted on *Ae. japonicus* and therefore the first evaluation of its flight capacity. In the future, the flight propensity of *Ae. japonicus* determined in this study can be included as a parameter to model the colonization process of this invasive vector species.

*Aedes japonicus japonicus* (Theobald, 1901), also known as the East Asian bush mosquito, is an invasive species native to eastern Asian countries (Tanaka et al. 1979). This species invaded the United States of America in 1998, mainly via passive dispersal, through the trade of used tires (Andreadis et al. 2001).

Europe is also concerned by the spread of this mosquito since it was first detected in 2000 in France (Schaffner et al. 2003) and then in 2002 in Belgium (Versteirt et al. 2009). The East Asian bush mosquito then colonized Austria, Bosnia-Herzegovia, Croatia, Germany, Hungary, Italia, Lichtenstein, Luxembourg, the Netherlands, Serbia, Slovenia, and Spain (Becker et al. 2011, Kampen et al. 2012, Ibañez-Justicia et al. 2014, Kampen and Werner 2014, Seidelet al. 2016a, b; Eritja et al. 2019, Robert et al. 2019, Schaffner and Ries 2019, Janssen et al. 2020). The presence of *Ae. japonicus* was recorded in 2013 in the northeast of France (Krebs et al. 2014). Its distribution seems to follow an east to west axis as it was previously detected in Germany in 2008 (Becker et al. 2011, Kampen et al. 2012, Kampen and Werner 2014). Thus, the distribution of *Ae. japonicus* has greatly expanded geographically since it was first detected in Europe, suggesting great capacities of dispersal.

In addition to its invasive status, *Ae. japonicus* is also a potential vector of chikungunya, dengue, Japanese encephalitis, Saint Louis encephalitis, or West Nile virus (Rodhain 2008). Studying the flight capabilities of invasive vector species improves the understanding of their colonization process and the associated spread of vector-borne diseases. The expansion of species distribution is driven by the dispersal mechanisms and capacities of species. These are either passive or active means of dispersal (Wilkerson et al. 2021). Passive dispersal, for example, unintentional dispersal by wind or human transport, is one of the common ways which leads to the introduction and propagation of invasive species in new areas (Service 1980, Crowl et al. 2008). On the other hand, active dispersal involves daily behaviors, such as active flying to reach suitable breeding or resting sites, to mate in swarms, to seek sugar sources or hosts (Service 1997, Verdonschot and Besse-Lototskaya 2014, Wilkerson et al. 2021).

Active dispersal of mosquitoes can be assessed in multiple ways, either in the field or in the laboratory, each method having its advantages and drawbacks. The most frequent method uses the Mark-Release-Recapture (MRR) technique. This technique consists of marking, with mostly fluorescent dyes, adult mosquitoes previously collected in the field or reared in the laboratory. Marked individuals are then released in a specific place in the study area around which traps have been installed in order to recapture them at different distances from the release point. Used in the field, MRR requires a strict experimental design to maximize the success of the recapture rate. Moreover, due to the inherent variability of field conditions, there is a lack of repeatability (Marini et al. 2010). The results of MRR experiments can be influenced by landscape structure, vegetation, meteorological conditions as well as other factors (Service 1997, Kerkow et al. 2019, Wilkerson et al. 2021). The physiology of mosquito species and their host preferences are parameters known to interfere with their behavior and therefore their active dispersal (Service 1997, Tsuda et al. 2008). Invasive vectors other than *Ae. japonicus* have been investigated through this MRR technique. For example, a MRR study in Australia on *Ae. aegypti* (Linnaeus, 1762) (Diptera: Culicidae) adults reared from a colony, powder-marked the mosquitoes in the laboratory before releasing them. The individuals were then recaptured using sticky traps (Muir and Kay 1998). A similar method was used on *Ae. albopictus* in Italy, but females were reared from wild eggs collected through a network of ovitraps (Marini et al. 2010). More recently, a MRR study using self-marking units marked mosquito adults with fluorescent pigments during the emergence process. Thus, all the steps (mark, release, and recapture) were done in the field (Vavassori et al. 2019).

Laboratory experiments under controlled conditions are the first step to reduce the variability of flight measurements and to evaluate the influence of a limited number of factors. A common experimental design is tethered flight techniques, which have been used for more than 50 yr (Minter et al. 2018). Several devices to study flight capacities exist, such as wind tunnels, free flight chambers, and tethered flight systems, also referred to as rotational flight mills (Minter et al. 2018).

A rotational flight mill is a device composed of a rotational part, on which the insect is hung by the back of its thorax with an adhesive, and a static part (Minter et al. 2018). In order to reduce friction forces, the static part of the flight mill is generally composed of either sapphire bearings (Rowley et al. 1968) or magnets (Martini et al. 2014). Due to the tarsal reflex phenomenon (Rowley et al. 1968), the mosquitoes are compelled to fly when solid surfaces are removed from under their legs. Thus, the rotational flight mill can be set in motion and the flight can be recorded by infrared, light or magnetic sensors, or by video (Minter et al. 2018, Spitzen and Takken 2018). Flight propensity (distance, duration, and velocity) can be assessed by tethered flight under laboratory conditions to test several parameters, physiological or environmental, which could influence flight behavior (Minter et al. 2018). Flight mill studies have been conducted on various insect species such as bumblebees (Hymenoptera) (Kenna et al. 2019), beetles (Coleoptera) (Attisano et al. 2015), or various true bugs (Heteroptera) (Martini et al. 2014, Hahn et al. 2017), including vectors like black flies (Simuliidae) (Diptera: Simuliidae) (Stanfield and Hunter 2010) or kissing bugs (Triatominae) (Heteroptera: Triatominae) (Castro et al. 2014). Regarding mosquitoes (Culicidae), the flight capacity of many species has been evaluated using flight mill devices: *Culex tarsalis* (Coquillett, 1896) (Rowley 1970), *Aedes vexans* (Meigen, 1830) (Briegel et al. 2001b), *Anopheles gambiae* (Giles, 1902) (Kaufmann and Brown 2008), *Anopheles stephensi* (Liston, 1901) (Schiefer et al. 1977), *Ae. albopictus* (Briegel and Timmermann 2001) and *Ae. aegypti* (Rowley and Graham 1968, Briegel et al. 2001a, Bargielowski et al. 2012). However, to our knowledge, there is a significant lack of knowledge on the dispersal capacities of the East Asian bush mosquito, *Ae. japonicus*, both in the field (*e.g.* MRR) and under laboratory settings (*e.g.* flight mills).

In this study, we investigated for the first time the flight capabilities of *Ae. japonicus* using a rotational flight mill under controlled laboratory conditions. First, we combined the flight mills used in previous studies to optimize the design of our device in order to minimize both the friction forces and the weight carried by the mosquitoes. Then, we used the flight mill to record the time, the distance, and the speed of flight of *Ae. japonicus* females according to their age.

## Material and Methods

### Mosquito Collection

Larvae of *Ae. japonicus* were collected from Reichstett, a city located approximately 7 kilometers north of Strasbourg (48.64°N, 7.75°E). The area consisted of a mixture of forest and family gardens bordering a residential area and a cemetery. Larvae were collected from various artificial containers such as rain barrels, plastic buckets, or outdoor bathtubs from the end of July to mid-October. The larvae were then reared to adults in mosquito breeders (Bioquip, Rancho Dominguez, CA). These breeders consisted of two polystyrene jars screwed to a central plastic lid. These contained a vinyl funnel through which emerging adults flew from the bottom jar (water sample with larvae and pupae) into the upper jar. After emerging, adults of nulliparous and nonblood-fed females were grouped according to age. Age categories were defined as 0, 1, and 2-wk-old, and correspond to females aged 1, 7, and 14 d old, respectively. As emergence was reported once per day, a 24-h period before and after these thresholds was selected. Accordingly, group A consisted of mosquitoes who had emerged early, up to 48 h. Mosquitoes aged 6 to 8 d were included in group B while mosquitoes aged 13 to 15 d were included in group C. Females were reared in groups of maximum 10 individuals in breeders, at constant temperature (25.1°C ± 1.8) and constant relative humidity (64 ± 4 %) and fed on a 10% sugar solution.

### Flight Mill Device

**Fig. 1.**
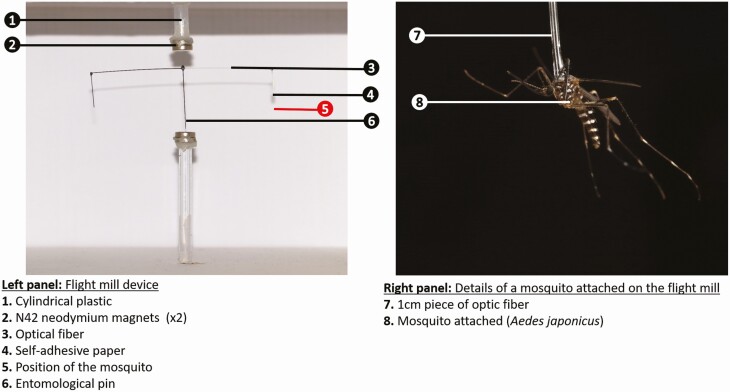
Flight mill system.

To minimize friction force, we combined previous designs (Martini et al. 2014, Attisano et al. 2015) to build a new flight mill. Our design used a magnetic system and optic fiber as super light materials (Fig. 1). The magnetic bearing was made of two cylindrical parts made of plastic, each with two glued neodymium magnets N42 (Master Magnetics Inc., Castle Rock) at the tip. A 11-cm-long midsection piece of optic fiber was glued perpendicularly to the tip of an entomological pin (size 2). At each end of the optic fiber, an extra piece measuring 1 cm was added parallel to the entomological pin. This assemblage between both magnets served to stabilize the pivoting arm of the device. Prior to the experiment, 35 females of *Ae. japonicus* were weighed together in triplicate (20 mg ± 1) to determine the weight of the necessary counterpart made of optic fiber for a single mosquito. Given that a piece of optic fiber measuring 1 cm is used to glue the mosquitoes to the pivoting arm, and considering the weight of a female *Ae. japonicus* (0.57 mg), the counterpart was determined to be a 2-cm-long piece of optic fiber. Half of the pivoting arm was colored in black to improve the sensitivity of the automatic detection of the camera system.

The mosquito was glued by the thorax to a 1-cm-long optic fiber with wood glue (Pritt, Henkel France S.A.S, Boulogne Billancourt, France). The fiber was subsequently bound to the pivoting arm using a piece of self-adhesive paper. The absence of toxicity of the wood glue on the lifespan of mosquitoes was evaluated beforehand by gluing a piece of optic fiber on the thorax and freeing the mosquitoes in rearing cages. No difference in the mortality of adults was observed between the mosquitoes with wood glue and without glue for 48 h (Supp Table 1 [online only]).

### Video Recording of Mosquitoes

The flight of mosquitoes was recorded by a PiNoiR Camera v2 (Raspberry Pi Foundation, Cambridge, England) mounted on a Raspberry Pi3 model B+ (Raspberry Pi Foundation). Temperature and humidity were recorded at the beginning of the flight.

**Fig. 2.**
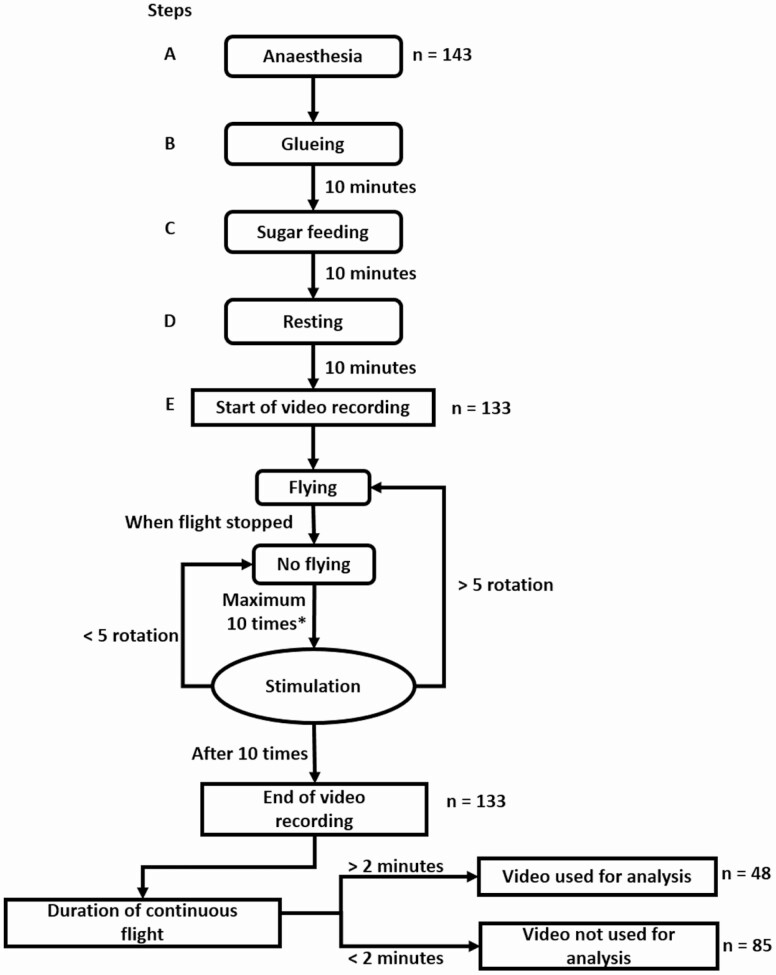
Diagram of the recording protocol of the flight mill experiment. The « flying » behavior was determined as a continuous flight for more than 5 revolutions of the pivoting arm. The « no flying » behavior was determined in the case of either more than 10 stimulations or 10 min without flying.

The diagram of the complete protocol, from mosquito preparation to video recording, is represented in Fig. 2. Mosquitoes were anesthetized on a cool box filled with ice for 15 min and glued according to the protocol described above (Fig. 2—steps A–B). After 10 min to let the glue dry, a 10% sucrose solution was proposed to the mosquito using soaked cotton for another 10 min (Fig. 2—step C). The mosquito was eventually left to rest for 10 more min (Fig. 2—step D).

Before the flight, the relative volume of the abdomen, corresponding to an approximation of sugar intake, was recorded based on a scale ranging from 1 to 4: one corresponds to no sugar intake (empty abdomen), and four, a maximal sugar intake (full abdomen).

Nulliparous females were tested under constant light condition (L:D 14:10) at a temperature ranging between 21.4°C–28.8°C, and RH of 56–74%, both parameters being recorded before the flight.

After the 10 min resting step, the pivoting arm attached to the mosquito was placed between the magnetic bearings. Removing the supporting structure from the legs triggered the flight reflex (Rowley et al. 1968) and the female was compelled to fly until exhaustion. Video recording started as soon as the flight began. One video file was generated per individual (Fig. 2—step E). Stimulation, to enhance flight reflex, was performed with a piece of plastic which was quickly put under the legs of the mosquito and removed.

### Video Analysis

A total of 48 videos lasting longer than 10 min, in which the mosquitoes flew for at least 2 min, were analyzed. For each individual, parts of the video with more than 2 min of continuous flight were analyzed with a video tracking software, ToxTrac (Rodriguez et al. 2018). The software gave the duration and distance of flight. However, the ToxTrac software had difficulty in detecting and analyzing mosquito flight for continuous flights lasting less than two minutes. These occurred frequently because stimulations were required and disrupted the tracking. Therefore, all video segments were also analyzed manually under the BORIS software (Friard and Gamba 2016). The duration of flight and the number of turns were collected. The distance was calculated based on the number of turns and the length of the optic fiber of the pivoting arm, as the flight of the mosquito was limited to a circle with a perimeter of 34.54 cm in length. Videos analyzed under BORIS and ToxTrac were summed per individuals. The average speed was calculated as the ratio between distance and duration.

Data of duration of flight (in seconds ‘s’), distance (in meters ‘m’), and speed (in meters per seconds ‘m.s^–1^’) were analyzed for each specimen, according to its age (Group A, group B, group C). Each group was represented by 16 females at the end of the study.

### Statistical Analysis

All statistical analyses were performed with the R language (R Development Core Team 2009). Two-tailed tests were performed with error type I α = 5%. Mean and median results were expressed as mean ± standard deviation (SD) or as median ± interquartile range (IQR).

Video selection regarding temperature, humidity, and sugar feeding were analyzed with Wilcoxon-Mann-Whitney tests.

Flight parameters were analyzed for each group of females with Kruskall-Wallis tests.

Factor Analysis of Mixed Data (FAMD) was used to analyze correlation between flight propensity of mosquitoes (duration, distance, and speed), age, temperature, and relative humidity as well as the group and sugar intake.

## Results

In total, 143 females were anesthetized and 133 successfully achieved the flight protocol. Ten specimens were unable to fly when placed on the device due to problems during the gluing step. These were either caused by a lack of anesthesia which led to the presence of glue on the wings (*n* = 6), or by a lack of glue that allowed the specimen to fly away (*n* = 4). Among the 133 videos, 85 were not used for analysis since: (i) the mosquito showed no flight behavior during 10 min despite stimulations; or (ii) the mosquito showed a minimal flight propensity that was not adapted for analysis due to a high number of human interruptions to stimulate the mosquito during the 10-min period. Therefore, flight parameters (distance, duration, and speed) were determined based on a total of 48 videos, each group (A–C) being represented by 16 videos.

**Fig. 3.**
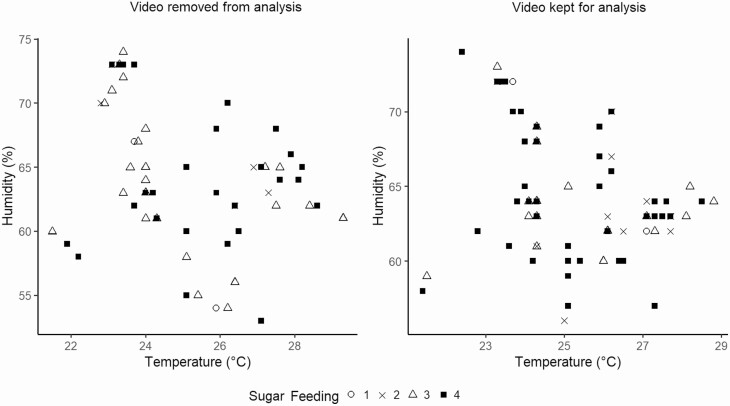
Influence of temperature, humidity and sugar feeding on video selection. None of these parameters were significant.

Neither temperature (W = 2344, *P* = 0.5337) nor humidity (W = 2331.5, *P* = 0.4998) had an impact on the selection of the chosen videos (Fig. 3—A). Sugar feeding, corresponding to the intake of sugar solution prior to flight, was not a factor in the selection of videos either (W = 2354, *P* = 0.4172) (Fig. 3—B).

**Fig. 4.**
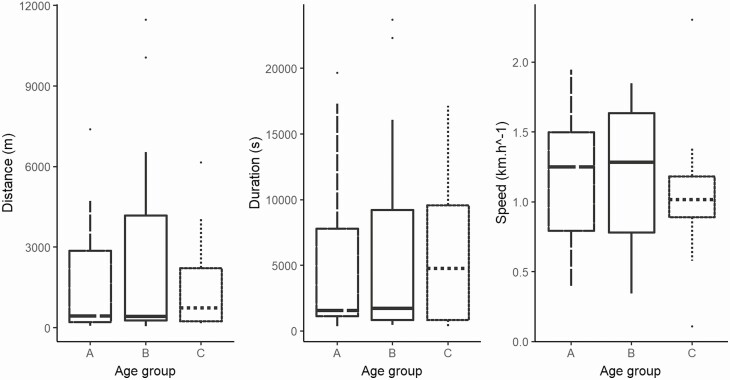
Distance, time and speed of *Ae. japonicus* flight. Groups are set according to the age of females. Group A consisted of females aged 24 to 48 h after emergence. Group B is composed of females aged 6 to 8 d old while group C includes females aged 13 to 15 d old.

FAMD appeared to show discrimination between groups but the percentage of variance was too low to be significant (less than 50%: dimension 1 = 23.1%; dimension 2 = 20.9%). This observation was confirmed by the independent Kruskal-Wallis test which showed no difference between the three groups regarding speed (df = 2; χ ^2^ = 1.1985; *P* = 0.5492), duration (df = 2; χ ^2^ = 0.24691; *P* = 0.8839) nor distance (df = 2; χ ^2^ = 0.39207; *P* = 0.822) (Fig. 4). Therefore, the data collected for each female, whatever its group, were merged (*n* = 48) for further analysis. Table 1 gathered the main characteristics of flight propensity for the whole dataset (*n* = 48). On average, half of the females flew 438 m, in 28 min, at a speed of 1,120 m.h^–1^. We recorded a maximum flight distance of 11,466 m, lasting 6 h 35 min, for a single female aged 7 d after emergence, fully engorged with sugar at 24.2°C and 60% RH. Regarding speed, a maximum of 2,300 m.h^–1^ was recorded for an older female aged 15 d after emergence, at 27.7°C and 62% RH, for a flight lasting 14 min, though her sugar feeding score was 2 out of 4. In contrast, we recorded a minimum flight of 5 min 37 s, for a distance of 43.09 m, for another female aged 6 d after emergence, at 23.7°C and 72% RH, which had badly fed on sucrose (1 out of 4 on the sugar feeding scale). The slowest mosquito, aged 15 d after emergence, flew at 10.7 m.h^–1^ on average during 2 h 5 min, with a 3 out of 4 sugar feeding score, at 24.1°C and 63% RH.

**Table 1.** Flight propensity of female *Ae. japonicus*

*n* = 48	Duration of flight (s/ h:m:s)	Distance (m)	Speed (m.h^–1^)
Minimum	337/ 00:05:37	43	107
Maximum	23,696/ 06:34:56	11,466	2,303
Mean	5,838/ 01:37:18	1,980	1,129
Standard-deviation	6,510/ 01:48:30	2,708	468
First quartile	916/ 00:15:16	243	839
Median	1,674/ 00:27:54	438	1,123
Third quartile	9,243/ 02:34:03	2,627	1,428
90%	16,062/ 04:27:36	6,155	1,740

**Fig. 5.**
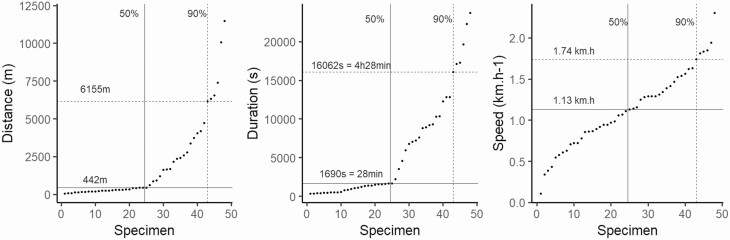
Ascending data of flight propensity. For each parameter recorded, data of females are sorted in ascending order. The 50% and 90% values are plotted in light grey for each parameter.

**Fig. 6.**
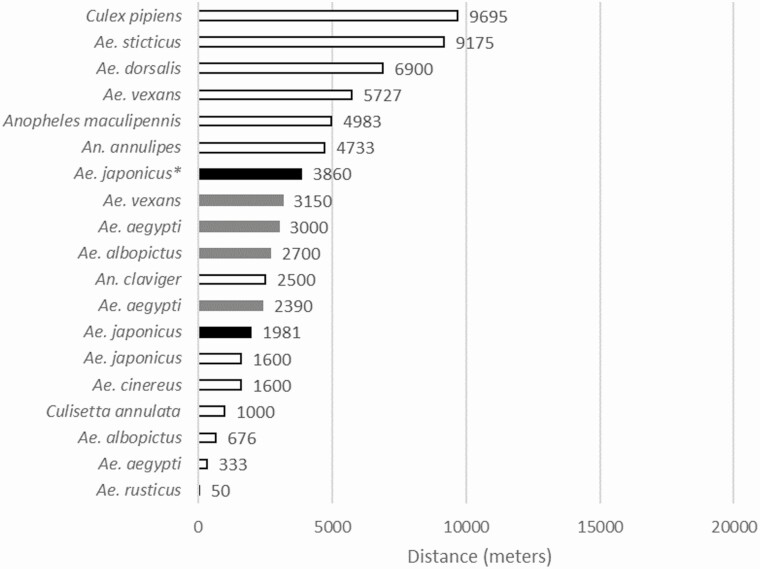
Comparison of flight capacities of *Ae. japonicus* and other species. Each solid bar represents the reported distance given by a specific study for a species, meaning that a single species can be represented by multiple bars/studies. Both results from this study are shown in black, the asterisk corresponds to the mean value obtained after removing females that flew less than 500 m. Data from the literature review of Verdonschot and Besse-Lototskaya (2014) are shown in light grey. Additional data from previous flight mill experiments are shown in medium grey (Briegel et al. 2001a,b; Bargielowski et al. 2012, Kaufmann et al. 2013).

Data of distance, duration, and speed were plotted and sorted according to ascending order to visualize the capacities of each individual (Fig. 5). With regards to the general shape of the data, distance and duration showed an exponential-like tendency suggesting a great variation between weak flyers and specimens exhibiting great dispersal capacities. On the contrary, speed grew linearly, highlighting the correlation between distance and duration of flight. Regarding females with stronger capacities, 10% of females flew: (i) more than 6,155m, (ii) during 4 h 28 min and, (iii) at a speed of 1,700 m.h^–1^ (greyish areas in Fig. 5). In order to compare our results for distance with other species, we plotted the mean distance of flight of *Ae. japonicus* from this study and those available for various species also found in the northeast of France (Briegel et al. 2001a, b; Bargielowski et al. 2012, Kaufmann et al. 2013, Verdonschot and Besse-Lototskaya 2014) (Fig. 6). We plotted the average flight recorded by the flight mill devices, despite the fact that the various studies did not use the same protocol (variation of data recorded, variation of threshold between the analyzed specimens, or duration of observations). The selected species showed flight capacities ranging between 50 m and almost 10 km. Many *Aedes* species, including *Ae. japonicus*, flew less than 5 km.

## Discussion

Though it has never been deeply investigated, *Ae. japonicus* is considered as an invasive vector mosquito capable of great capacities of dispersal. This study evaluates for the first time the flight capacities of *Ae. japonicus* females under laboratory conditions using a flight mill device. The results showed a mean capacity of 1,980 m at 1,129 m.h^–1^ and 10% of the females tested were able to reach more than 6,155 m at 1,700 m.h^–1^. When removing the data of females flying less than 500 m to fit the methodology of a previous flight mill study on *Ae. albopictus* (Kaufmann et al. 2013), our results reveal that *Ae. japonicus* is a stronger flyer than *Ae. albopictus,* with 3,860 m (*n* = 23) in comparison to 3,000 m, respectively.

Even though the number of females tested per group (*n* = 16) is relatively low, our results are still comparable with similar studies. In comparison, the number of observations in the study of Bargielowski et al. (2012) was represented by 21, 14, 26, 14, and 24 individuals. Kaufman et al. (2013) also tested 20 females of *Ae. albopictus* per condition, resulting in groups composed of 14 to 19 individuals in the analyzed dataset.

Many authors have studied the flight capacities of mosquitoes in past studies. These analyze cumulative flights over several hours (16 h to 20 h) and therefore flight patterns are part of the data recorded (Briegel et al. 2001a,b; Bargielowski et al. 2012, Kaufmann et al. 2013). We chose to record only the first flight until exhaustion, therefore no flight pattern can be assessed. In addition, we chose to remove females depending on their behaviors on the flight mill, whereas many authors have used a distance of 500 m (Bargielowski et al. 2012, Kaufmann et al. 2013) or 1,000 m (Briegel et al. 2001b) as a threshold. Among the total of specimens tested, only 36.1% were used for analysis. This number is similar to another study on Ae. *aegypti* (36.8%, ranging from 29.8% to 38.2% for the wild type) (Bargielowski et al. 2012) but lower than the study of Briegel et al. (2001b) on *Ae. vexans* (77.6%).

Flight mill flying behaviors usually differ from free flight behaviors (Attisano et al. 2015). Like other studies using rotational flight mills, we took into account two major drawbacks to design our own device. First, the mosquito on the device does not have to generate the same lift required in free flight. As such, it is no longer required to support its weight and this can artificially increase its dispersal capacities (Minter et al. 2018). To minimize this effect, we chose to add a counterpart, whose weight was similar to a mosquito, on the arm of the rotational flight mill. Second, the mosquito needs to produce enough energy to start moving the arm of the mill (Minter et al. 2018). This energy was minimized in the present study by using optical fiber as a super light material.

This methodology, which uses a flight mill device and controls parameters in a laboratory setting, also has an influence on the reported distance. In the field, temperature and relative humidity influence flight capacities. Optimum flight activity has been evaluated to be approximately at 27°C and 80% RH (Rowley and Graham 1968). Environmental factors can also influence the population dynamics of vector species (Rodhain 2008). Climate change can modify environmental conditions, which can lead to favorable conditions for vector species and therefore to the spread of vector-borne diseases (Duvallet 2015). Dispersal of vectors and pathogens is also influenced by the migration of humans and the transport of merchandise (Schaffner and Karch 2000, Duvallet 2015).

In the literature, mosquitoes are reported to fly between 50 m and 50 km, depending on the various conditions and species (Verdonschot and Besse-Lototskaya 2014). Specimens of the *Aedes* genus are generally classified as weak flyers (<50 m) or strong flyers (>4,000 m) (Verdonschot and Besse-Lototskaya 2014). For example, MRR studies on species within the *Aedes* genus reported a distance of 55.6 m, while the distance increased to 12,441 m with the use of a flight mill device (Verdonschot and Besse-Lototskaya 2014). *Ae. albopictus,* another invasive species, was reported to fly 75 m in an MRR study (Verdonschot and Besse-Lototskaya 2014) while in others, adults regularly flew more than 250 m (Marini et al. 2010, Vavassori et al. 2019).

Regarding the East Asian bush mosquito, Seidel et al. (2016b) reported a migration of 100 km over the 7 yr of colonization of *Ae. japonicus* in Hungary, corresponding to a dispersion of 14.3 km per yr. More recently, another study conducted in Hungary from 2017 to 2018 showed that *Ae. japonicus* expanded its distribution northeast by 180–200 km from the first locality recorded in 2012 (Sáringer-Kenyeres et al. 2020). This amounts to an estimated potential dispersion of 30–33 km per year. These results at the population level make it impossible to distinguish between passive and active dispersal, and individual capacity. However, this study underlines the great dispersal capacity of *Ae. japonicus*. Based on records made by Eritja et al. (2021), *Ae. japonicus* distribution expanded around 3,000 km^2^ per year during the 2018–2020 period in Spain. As these authors suggested, natural dispersal is likely to happen through riparian corridors rather than road corridors, though the means of introduction of this species remain unknown. In Switzerland, results from an ovitrap network also show great dispersal capacities for *Ae. japonicus* between 2013 and 2018, and as suggested by the authors, these results favor an active dispersal (Müller et al. 2020). At the individual level, it was reported that *Ae. japonicus* is capable of flying an average maximum distance of 1.6 kilometers (Moberly et al. 2005, Verdonschot and Besse-Lototskaya 2014). In their meta-analysis of mosquito flight capacities (Verdonschot and Besse-Lototskaya 2014), the authors reported that *Ae. japonicus* had capacities of 1.6 km from Moberly et al. (2005) (Verdonschot, personal communication). However, Moberly et al. (2005) cited this maximum distance erroneously when referring to the work of Fonseca et al. (2001) though the latter did not mention such a distance in their study. Comparatively, we found that 37.5% (18 out of 48) of females flew well beyond this distance, with a maximum of 11,466 m, corresponding to almost seven times more. Though flight mill conditions may artificially increase the range of dispersal, our study shows that *Ae. japonicus* is capable of flying several kilometers, which may explain its patchy distribution pattern.

The influence of age varies depending on the species. In another flight mill study by Nayar and Sauerman (1973), mosquitoes were tested individually twice a week during their life-span. *Cx. nigripalpus* (Theobald, 1901) flew at 1 km per h during a 10 consecutive week period, while *Aedes sollicitans* (Walker, 1856) (Diptera: Culicidae) flew at 1.2 km per h during 5 consecutive weeks. In another study by Rowley and Graham (1968), right after emergence, females of *Aedes aegypti* flew less than 500 m in a flight mill. The flight capacities of this species increased to 6,354 m 24 h after emergence and the maximum flight capacity of *Ae. aegypti* was 9,108 m for 2-wk-old females, followed by decreasing flight capacities until the end of the experiment, at 6 wk. As such, the study of Rowley and Graham (1968) showed that flight capacities increase during the first two weeks and decline after. In addition, Rowley and Graham (1968) reported a great individual variation in flight capacities after recording a maximum distance of 17,392 m for *Ae. aegypti*. Like flight distance, speed of flight in their study decreases after the mosquitoes are aged 2 wk. Our results for *Ae. japonicus* tend to be similar: the maximal dispersal capacities were found for individuals which were approximately 1 wk old. It would seem that postemerging females (aged less than 48 h) and older ones (approximately 2 wk old) showed lower dispersal capacities, though this is not statistically supported. Further investigations with a higher number of mosquitoes tested are required to confirm this difference. Furthermore, wing abrasion is reported to be an age factor that reduces flight capacities, as shown in the species *Mansonia titillans* (Walker) (Diptera: Culicidae) with specimens older than 6 wk (Nayar and Sauerman 1973). In the present study, we also observed wing abrasion on females aged 13 to 15 d which could explain the slight difference of flight observed between these females and younger ones. Damaged wings may reflect physiological decline as females get older. In our study, wing abrasion may have been hastened by the contact with the plastic walls of the breeders and the closeness of specimens with each other. In addition, body size is known to influence dispersion, as females with bigger wings show greater flight capabilities (Faiman et al. 2020). In the present study, females exhibited a similar body size and were reared in similar conditions, though specimens were not measured. Recently, in thermal experiments, Reuss et al. (2018) showed that wing-length of *Ae. japonicus* is correlated to body size. Thus, future studies on *Ae. japonicus* whose objective is to investigate the effect of body size on a biological trait could measure the wing-length parameter, as it is a good estimate of body size.

The reproductive status, in particular the distinction between blood-fed or gravid females, is also a parameter known to influence dispersal. Huestis et al. (2019) captured four times as many females than males, and 90% of them had taken a bloodmeal. Similarly, Faiman et al. (2020) tested wild females on a tethered flight device, and 92% of them were gravid. These authors reported that gravid females showed a higher rate of flight activity than unfed females, when sampling was enough to perform statistical tests. Laboratory colonization of *Ae. japonicus* is reported to be complex, critical steps include successful mating and blood-feeding (Williges et al. 2008; Hoshino et al. 2010). When laboratory colonization was successful, blood-feeding was performed on anesthetized animals (birds or mice) during the first generations. In parallel to this study, we tried to rear *Ae. japonicus* in laboratory though our attempts were unsuccessful. Both mating and artificial blood-feeding (no anesthetized animals available) were unsuccessful and therefore both gravid and blood-fed conditions were not investigated in the present study.

Among the existing techniques to study flight dispersal, MRR has the advantage of allowing the study of active dispersion in the field. Due to the wide range of possibilities and dyes to mark specimens, MRR makes it possible to study a high number of individuals and several cohorts or species at the same time (Reisen et al. 2003). In addition, to measure the effective distance flown from the release point to the collection site, the direction, and the dispersal rate can be evaluated with this technique (Cho et al. 2002, Russell et al. 2005). However, the recapture percentage of MRR is low, and therefore many individuals need to be processed to compensate the low rate. Moreover, though this technique is suitable for fieldwork, MRR is sensitive to environmental conditions, and particularly wind which plays a major role (Reisen et al. 2003, Verhulst et al. 2013). Recently, a comparison study on CDC traps succeeded in determining a combination of attractants used to increase the attractiveness of the traps for collecting *Ae. japonicus* mosquitoes. This study offers a useful tool that can be used in surveillance programs of this species (Balestrino et al. 2016). This trapping methodology may be applied in a future MRR study of *Ae. japonicus* to provide useful field data on its dispersal capacities.

Temperature and species distribution are the usual parameters used in models of population dynamics (Cailly et al. 2012, Tran et al. 2013) and to estimate dispersal capacities (Lutambi et al. 2013). Models can be adjusted with various factors and used to predict future distribution of *Ae. japonicus* (Cunze et al. 2019, Peach et al. 2019). Precision of these models increases when additional parameters such as wind, landscape (Kerkow et al. 2019), and life history traits of the biological model (Wieser et al. 2019) are taken into account. Whether obtained in field or laboratory conditions, data on dispersal capacities are one of the parameters which should be implemented in mathematical models to map the spread of invasive species and make predictions. Future modeling studies can use the flight capacity of *Ae. japonicus* reported in our study as an additional parameter to improve the description of the colonization process of this invasive vector species.

## Supplementary Material

ieab093_suppl_Supplementary_Material
