# Supplementary material for: Laboratory Evaluation of Flight Capacities of Aedes japonicus (Diptera: Culicidae) Using a Flight Mill Device
Source: J Insect Sci. 2021 Dec 4;21(6):18. doi: 10.1093/jisesa/ieab093 (PMC8643834; doi:10.1093/jisesa/ieab093)
Supplement: ieab093_suppl_Supplementary_Material [file ieab093_suppl_supplementary_material.docx]

Supplementary material

Supplementary material 1. Table 1. Influence of glue on the mortality rate of *Ae. japonicus* females, 24 hours and 48 hours after gluing.

| Type | Glued females | Non-glued females |
| --- | --- | --- |
| n | 97 | 97 |
| Mortality after 24 hours (%) | 1,83 ± 2,13 | 1,0 ± 2,0 |
| Mortality after 48 hours (%) | 3,61 ± 3,30 | 1,78 ± 2,09 |

Wilcoxon test (48 hours): W=9.5; *P*= 0.7389

Wilcoxon test (48 hours): W=10.5; *P*= 0.5516
